# Dateninfrastrukturen für die Gesundheitsforschung: Ethische Rahmenbedingungen und rechtliche Umsetzung

**DOI:** 10.1007/s00103-022-03648-2

**Published:** 2023-01-16

**Authors:** Christoph Schickhardt, Eva Winkler, Ulrich Sax, Benedikt Buchner

**Affiliations:** 1 Sektion Translationale Medizinethik, Nationales Centrum für Tumorerkrankungen (NCT) Heidelberg, Deutsches Krebsforschungszentrum (DKFZ) Heidelberg, Heidelberg, Deutschland; 2 Sektion Translationale Medizinethik, Abteilung Medizinische Onkologie, Nationales Centrum für Tumorerkrankungen (NCT) Heidelberg, Universitätsklinikum Heidelberg, Heidelberg, Deutschland; 3 Institut für Medizinische Informatik, Translationale Verbundforschung, Universitätsmedizin Göttingen, Göttingen, Deutschland; 4 Lehrstuhl für Bürgerliches Recht, Haftungsrecht und Recht der Digitalisierung, Universität Augsburg, Universitätsstr. 24, 86159 Augsburg, Deutschland

**Keywords:** Verantwortlichkeit, Einwilligung, Forschungsprivileg, Gesundheitsdaten, Betroffenenrechte, Accountability, Consent, Research privilege, Health data, Rights of the data subject

## Abstract

Die Rolle von Dateninfrastrukturen für die Gesundheitsforschung beschränkt sich nicht allein darauf, als Service oder Schnittstelle für den Datenaustausch zwischen Datenerzeuger:innen und -nutzer:innen zu fungieren. Die Infrastruktur ist vielmehr selbst ein Akteur im Prozess des Datenteilens, der für diesen auch entsprechend Verantwortung trägt.

Dies gilt zuallererst für die Rechtmäßigkeit der Verarbeitung personenbezogener Daten. Fußt die Datenverarbeitung auf einer Einwilligung der betroffenen Person, ist auch seitens der Infrastruktur sicherzustellen, dass sämtliche Datenverarbeitungsprozesse von dieser Einwilligung erfasst sind. Stützt sich die Datenverarbeitung auf einen gesetzlichen Erlaubnistatbestand, müssen im Rahmen der Infrastruktur v. a. technische und organisatorische Maßnahmen ergriffen werden, damit ein möglichst hohes Datenschutzniveau sichergestellt ist. Zudem fällt der Infrastruktur eine Verantwortung zu, wenn es um die Umsetzung von Betroffenenrechten wie Auskunft, Berichtigung oder Löschung von Daten geht und um den Umgang mit Zufalls- und Zusatzbefunden.

Die Frage, mit welchem Selbstverständnis sich Forscher:innen in Infrastrukturprojekte einbringen und in welcher Form auch privatwirtschaftliche Unternehmen in solcherlei Projekte eingebunden werden sollen, muss sich am Gemeinwohl orientieren. Damit einher geht die Verpflichtung, dass Infrastrukturen so weit wie möglich an den Prinzipien der Partizipation, der Transparenz und der Wissenschaftskommunikation ausgerichtet sind. Die Beachtung all dieser ethischen und rechtlichen Aspekte ist vor allem auch deshalb wichtig, weil nur auf diese Weise das Vertrauen aller Stakeholder gewonnen und damit die zentrale Basis für den erfolgreichen Aufbau und Betrieb einer Dateninfrastruktur geschaffen werden kann.

## Abstract (en)

The role of data infrastructures for health research is not limited to acting as a service or interface for data exchange between data producers and data users. Rather, the infrastructure itself is an actor in the process of data sharing and therefore also bears responsibility for this process.

This applies first of all to the lawfulness of personal data processing. If data processing is based on the consent of the data subject, the infrastructure must also ensure that all data processing is covered by this consent. If the data processing is based on a statutory basis, the infrastructure must ensure the highest possible level of data protection, in particular through technical and organizational measures. In addition, the infrastructure is also responsible for implementing the rights of data subjects, such as the right to information, rectification or erasure of data, and dealing with incidental or additional findings.

The question of how researchers regard their involvement in infrastructure projects and how private companies should be involved in such projects must be based on the principle of public welfare. This is accompanied by the obligation of infrastructures to take into account the principles of participation, transparency, and scientific communication as far as possible. Observing all these ethical and legal aspects is especially important because only by doing so can the trust of all stakeholders be established and thus the central basis for the successful construction and operation of data infrastructures be provided.

## Einleitung

Die nachfolgenden Ausführungen benennen ethische und rechtliche Aspekte von Dateninfrastrukturen für die Gesundheitsforschung, wie sie derzeit insbesondere im Rahmen des Aufbaus einer Nationalen Forschungsdateninfrastruktur (NFDI) gefördert werden. Die Ausführungen verstehen sich nicht als wissenschaftlich tiefgehender Grundlagentext, sondern als Werkstattbericht aus der Praxis für die Praxis. Ziel ist es, Überlegungen zu normativen Aspekten auszuformulieren und all jene, die in einem NFDI-Konsortium oder anderen Zusammenhängen mit dem Aufbau einer Infrastruktur beschäftigt sind, zu informieren.

Der Artikel benennt aus vorrangig ethischer Perspektive zu beachtende Aspekte und Leitlinien und zeigt aus rechtswissenschaftlicher Perspektive die thematisch entsprechende rechtliche Lage und rechtlichen Umsetzungsmöglichkeiten auf. Dabei verlaufen die Grenzen zwischen den ethischen Leitlinien und den rechtlichen Aspekten teilweise. Dieses transdisziplinäre Vorgehen, die beiden normativen Perspektiven eng zu verzahnen, entspricht dem eher pragmatischen Charakter des Artikels und spiegelt die enge Kooperation zwischen Vertreter:innen von Ethik und Recht wider, wie sie in der täglichen Projektarbeit, z. B. in Arbeitsgruppen zu ethischen, rechtlichen und sozialen Aspekten (ELSA), zu Themen wie Datenschutz und normativer Governance zu finden ist.

## Ausgangspunkt: Spezifische Verantwortlichkeit einer Dateninfrastruktur

Auf Vorschlag des Rats für Informationsinfrastrukturen (RfII) einigte sich die Gemeinsame Wissenschaftskonferenz (GWK) im Jahr 2018 darauf, im Rahmen einer Nationalen Forschungsdateninfrastruktur (NFDI) den Aufbau von Konsortien zu fördern und zu koordinieren, mit denen die Wissenschaftscommunitys wissenschaftsgetrieben für ihre jeweiligen Anforderungen passende Forschungsdateninfrastruktur etablieren können. In der ersten von der Deutschen Forschungsgemeinschaft (DFG) gesteuerten Runde wurden 9 Konsortien ausgewählt, die 2020 ihre Arbeit aufgenommen haben,^1^ darunter aus dem Bereich Biomedizin auch die Konsortien NFDI4Health und das Deutsche Humangenom-Phenomarchiv (GHGA).

Die Konsortien sollen Prozesse und Werkzeuge einer IT-gestützten Infrastruktur für die wissenschaftliche Forschung aufbauen. Damit stellt sich zum einen die Frage, welche Rolle und welche Verantwortlichkeiten den aufzubauenden Infrastrukturen in Zukunft zukommen werden. Zum anderen muss geklärt werden, welche Verantwortlichkeiten wiederum bei den Forscher:innen liegen, die Daten erzeugen und in die Infrastruktur einspeisen (im Folgenden Datenerzeuger:innen), oder bei den Forscher:innen, denen über die Infrastruktur Zugang zu und Nutzungsrechte an den Daten gewährleistet werden (im Folgenden Datennutzer:innen). Sind die Infrastrukturen einfach nur ein Service und eine Schnittstelle, die von den Datenerzeuger:innen und Datennutzer:innen eingesetzt werden, ohne eigene Verantwortlichkeiten für die Daten und die Rechte der Betroffenen? Oder kommt der Infrastruktur selbst eine Rolle als Akteur zu, die mit gewissen Verantwortlichkeiten für den Gang und die Verwendungen der Daten einhergeht, die über die Infrastruktur stattfinden? Die Fragestellungen erinnern an die regulatorischen Probleme, die wir aus der privatwirtschaftlichen Plattformindustrie des Internetzeitalters kennen.

Im Fall von Dateninfrastrukturen beschränkt sich deren Verantwortlichkeit jedenfalls nicht allein darauf, dass die Daten, die geteilt oder gemeinsam verwendet werden sollen, die sogenannten FAIR-Prinzipien (Findable, Accessible, Interoperable, Reusable) erfüllen. Trotz des sehr ethisch klingenden Namens handelt es sich bei diesen Prinzipien vorrangig um eher technische und organisatorische Anforderungen. Wenn die FAIR-Anforderungen erfüllt sind, *können* die Daten effektiv geteilt oder gemeinsam genutzt werden, weil dazu die technischen und organisatorischen Voraussetzungen bestehen. Die ethische und rechtliche Frage, wer unter welchen Bedingungen Zugang zu den Daten und Nutzungsrechte an den Daten erhalten darf und soll, ist damit aber noch nicht beantwortet.

Die Infrastrukturen haben neben der Aufgabe, Datenspeicherung anzubieten, die vorrangige Funktion, das Suchen nach und das Teilen von Daten für (sekundäre) Forschungszwecke zu ermöglichen. Das Teilen von Forschungsdaten betrifft die verschiedensten Parteien (Stakeholders): datenerzeugende Forscher:innen (inklusive forschende Ärzt:innen) und ihre Institutionen, potenzielle Datennutzer:innen und deren Institutionen, wissenschaftliche Zeitschriften, Forschungsförderer und Projektsponsoren, die geldgebende Allgemeinheit, aber eventuell auch die Privatwirtschaft (Unternehmen) und, sofern es um personenbezogene Daten geht, natürlich ganz besonders die Datensubjekte (Patient:innen oder Proband:innen), auf die sich die Daten beziehen. Ethisch verantwortungsvolles Datenteilen muss grundsätzlich die Rechte, die berechtigten Interessen, Anliegen und Sorgen aller betroffenen Stakeholder berücksichtigen und, wenn diese konfligieren, in einen gut begründeten Ausgleich bringen.

## Rechtmäßigkeit der Datenverarbeitung

Geht es um die gemeinsame Nutzung von (personenbezogenen) Forschungsdaten, stellt sich zuallererst die Frage, wer dafür verantwortlich ist, ob und wie externen Forscher:innen Zugang zu Daten gewährleistet wird. Bleibt die Verantwortung allein bei den Datenerzeuger:innen oder liegt sie bei der Infrastruktur? Wer hat in welcher Phase der Datenverarbeitung dafür Sorge zu tragen, dass personenbezogene Daten auf einer rechtmäßigen Basis erhoben, weitergegeben und für die verschiedensten Forschungszwecke weitergenutzt werden?^2^

Das Datenschutzrecht sieht für Konstellationen, in denen an einem Datenverarbeitungsprozess mehrere Verantwortliche beteiligt sind, das Konstrukt der gemeinsamen Verantwortlichkeit vor, geregelt in Art. 26 Datenschutz-Grundverordnung (DSGVO). Von einer gemeinsamen Verantwortlichkeit ist demnach auszugehen, wenn „zwei oder mehr Verantwortliche gemeinsam die Zwecke der und die Mittel zur Verarbeitung fest[legen]“. Unter Geltung der DSGVO kommt dem Rechtsinstitut der gemeinsamen Verantwortlichkeit eine erhebliche praktische Bedeutung zu, gerade auch für arbeitsteilige Datenverarbeitungsprozesse im Rahmen der medizinischen Forschung [1, S. 51].

Zwar soll das bloße Zurverfügungstellen von IT-Infrastruktur und deren Einsatz durch andere beteiligte Stellen noch nicht per se eine gemeinsame Verantwortlichkeit begründen [2 Rn. 65]. Bejaht wird eine gemeinsame Verantwortlichkeit jedoch dann, wenn im Zuge eines Forschungsprozesses mehrere Verantwortliche aufeinander abgestimmt und zeitgleich agieren; daher wird eine gemeinsame Verantwortlichkeit etwa auch schon dann angenommen, wenn ein Webseitenbetreiber seine Seiten für ein Forschungsprojekt zur Verfügung stellt und hierüber von den Projektbetreibern personenbezogene Daten erhoben werden [1, S. 52]. Darüber hinaus soll eine gemeinsame Verantwortlichkeit selbst dann noch in Betracht kommen, wenn personenbezogene Daten nicht zeitgleich, sondern nacheinander in einer Verarbeitungskette verarbeitet werden. Ausreichend ist insoweit, dass in einer Gesamtbetrachtung die Zusammenarbeit der verschiedenen Akteure notwendig ist, um den gewünschten Effekt (hier konkret das Teilen von Daten) zu erzielen [3 Rn. 22].

Geht man von einer gemeinsamen Verantwortlichkeit von Dateninfrastruktur und Forscher:innen aus, müssen diese in einer Vereinbarung transparent festlegen, wer welche Verpflichtungen nach der DSGVO erfüllt. Zu klären ist dann also, wer für das Einholen einer (wirksamen!) Einwilligung oder für die rechtskonforme Datenverarbeitung auf Grundlage gesetzlicher Erlaubnistatbestände verantwortlich ist. Gemäß Art. 26 Abs. 2 S. 1 DSGVO muss die Vereinbarung „die jeweiligen tatsächlichen Funktionen und Beziehungen der gemeinsam Verantwortlichen gegenüber betroffenen Personen gebührend widerspiegeln“. Dies bedingt, dass zwischen Infrastruktur und Forscher:innen die Rollen der jeweiligen Verantwortlichen klar definiert sind und die unterschiedlichen Datenverarbeitungsprozesse von allen Akteuren in ihren Einzelheiten präzise erfasst werden [4 Rn. 16].

### Schutz informationeller Selbstbestimmung: die Einwilligung

Der Schutz der informationellen Selbstbestimmung derjenigen, deren personenbezogene Daten verarbeitet werden, wird bis dato in der Praxis der Forschungsdatenverarbeitung in erster Linie durch den Erlaubnistatbestand der Einwilligung sichergestellt. Gesetzliche und ethische Grundlage der Verarbeitung von personenbezogenen Daten ist ganz überwiegend die (breite) Einwilligung seitens der betroffenen Personen [5, 6].^3^ Gerade zur Ermöglichung der Kernfunktion der Infrastrukturen, dass Daten für verschiedene Forschungsprojekte entdeckbar, nutzbar oder zur Verfügung gestellt werden (können), ist die breite Einwilligung (Broad Consent) das zur Zeit einzige auch in der Praxis umsetzbare Aufklärungs- und Einwilligungsmodell, während Alternativen wie Dynamischer Consent oder Meta Consent bisher eher theoretisch diskutiert werden und erhebliche praktisch-technische, datensicherheitsrelevante und ethische Herausforderungen implizieren [7, 8].^4^

Auf Grundlage einer breiten Einwilligung wird bestimmt, welche Arten der Datennutzung und Datenweitergabe durch die betroffene Person autorisiert werden. Alles, was mit den Daten in der Infrastruktur und vermittelt über die Infrastruktur geschieht, angefangen von der Speicherung bzw. dem Hochladen der Daten in die Infrastruktur, muss im Rahmen dessen bleiben, was durch die Einwilligung vorgegeben ist.

Im Sinne einer Rechtmäßigkeit der Datenverarbeitung und zur Erfüllung ihrer Rechenschaftspflicht (Art. 5 Abs. 2 DSGVO) muss auch seitens der Infrastruktur sichergestellt werden, dass sämtliche (geplante) Datenverarbeitungen von der Einwilligung der jeweils betroffenen Personen erfasst sind. Zu diesem Zweck sollte jedenfalls von den Datenerzeuger:innen die Garantie eingefordert werden, dass das Hochladen der Daten in die Infrastruktur und geplante Weitergaben (oder andere Verarbeitungen) von der Einwilligung gedeckt sind.

Für ein Mehr an Rechtssicherheit bietet es sich darüber hinaus aber auch an, dass seitens der Infrastruktur selbst – jedenfalls sporadisch, noch besser aber standardmäßig – bei jedem Hochladen von Daten geprüft wird, ob diese von der ursprünglichen Einwilligung gedeckt sind. Bei jeglicher Nutzung oder Ausleitung von Daten ist zudem sicherzustellen, dass die einst erteilte Einwilligung noch vorliegt und nicht mittlerweile widerrufen worden ist.

Über die Verpflichtung zur rechtmäßigen Datenverarbeitung hinaus können vonseiten der Infrastruktur auch noch unverbindlich für all jene Forscher:innen, die mit ihrer Datensammlung erst beginnen wollen oder offen für Veränderungen an ihren aktuellen Einwilligungsdokumenten sind, gute Aufklärungs- und Einwilligungstext-Modelle oder Textbausteine zur Verfügung gestellt werden (ggf. einfache bzw. altersgerechte Sprache, Textpassagen, Erklär-Grafiken). Mit derartiger Unterstützung können Infrastrukturen dabei helfen, möglichst gute Informations- und Einwilligungsprozesse zu entwickeln, die u. a. auch über die Nutzung der NFDI-Infrastruktur aufklären.

### Datenverarbeitung ohne Einwilligung: das Forschungsprivileg

Die Einwilligung mag als Legitimationsgrundlage für eine Datenverarbeitung durch Forschungsdateninfrastrukturen bislang oft im Zentrum stehen. Jedoch ist diese zentrale Rolle rechtlich nicht vorgegeben. Vielmehr steht der Maxime einer einwilligungsbasierten Datenverarbeitung – als Ausdruck informationeller Selbstbestimmung – die Freiheit der Forschung gegenüber, unter deren Schutz auch Dateninfrastrukturen als Rückgrat einer vernetzten und institutionenübergreifenden Forschung fallen.^5^

Beide Rechte, informationelle Selbstbestimmung und Freiheit der Forschung, müssen in einen Ausgleich miteinander gebracht werden. Verfassungsrechtlich ist vor allem im Bereich der Gesundheitsforschung bei der Gewichtung der Forschungsfreiheit auch auf den mit der Forschung erhofften Nutzen für die Allgemeinheit, d. h. das Gemeinwohl hinzuweisen [9], das aus ethischer Sicht hier sogar noch wesentlich schwerer wiegt als die Forschungsfreiheit der einzelnen Forscher:innen [10].

Der europäische Gesetzgeber hat den Konflikt zwischen informationeller Selbstbestimmung und Forschungsfreiheit dahingehend aufgelöst, dass er eine Datenverarbeitung zu Forschungszwecken grundsätzlich auch ohne Einwilligung der betroffenen Person zulässt. Kompensiert wird diese Privilegierung der Forschung in erster Linie dadurch, dass technische und organisatorische Maßnahmen ein möglichst hohes Niveau an Datenschutz und Datensicherheit garantieren müssen. Damit sind auch Dateninfrastrukturen in der ethischen und rechtlichen Pflicht, die Datenverarbeitungsprozesse so auszugestalten und abzusichern, dass der Eingriff in das informationelle Selbstbestimmungsrecht der betroffenen Personen so weit wie möglich reduziert wird.

Zu diesem Zweck steht Dateninfrastrukturen ein breit gefächertes Instrumentarium an möglichen Schutzmaßnahmen zur Verfügung, deren Einsatz bei den Datenerzeuger:innen und Nutzer:innen einer Infrastruktur gefördert werden kann, die aber auch bei den Infrastrukturen selbst eingesetzt werden können (vgl. dazu auch [11]). In Betracht kommen Instrumente wie die Anonymisierung, Pseudonymisierung oder Verschlüsselung von Daten ebenso wie die Etablierung geschlossener Datenräume und sogenannte Secure-Access-Lösungen (für eine gute Übersicht dazu s. a. [12]).

Eine wichtige Bedeutung können darüber hinaus auch Use-and-Access-Committees (UAC) spielen, die über die Herausgabe von Daten entscheiden und die Einhaltung von Regularien sicherstellen. Bei Entscheidungen über Zugang und Nutzung von Daten ist vor allem auch zu beachten, dass hierfür grundsätzlich nur Forschungsprojekte mit seriösem Methodendesign in Betracht kommen, die wichtige Forschungsfragen nicht nur aufwerfen, sondern diese auch potenziell beantworten können. Eine zentrale Beurteilungsmaxime ist darüber hinaus stets die durchgängige Risikominimierung zugunsten der betroffenen Personen.

## Kontrolle und Rückmeldung

Auch im Rahmen einer Datenverarbeitung durch Infrastrukturen muss jede betroffene Person (Patient:in oder Proband:in) die Möglichkeit haben, mittels Ausübung ihrer Betroffenenrechte die sie betreffende Datenverarbeitung kontrollieren zu können. Darüber hinaus geht es bei medizinischen Forschungsprojekten aber nicht nur um die klassischen Rechte wie Information oder Auskunfterteilung, sondern auch um die Frage, ob und wie die Kommunikation mit den Betroffenen im Falle von Zufalls- oder Zusatzbefunden zu gestalten ist.

### Kontrollrechte

Grundsätzlich ist jede Dateninfrastruktur – gemeinsam mit den Datenerzeuger:innen und -nutzer:innen – für die Umsetzung der Kontrollrechte der von der Datenverarbeitung betroffenen Personen verantwortlich. Sofern personenbezogene Daten gespeichert oder weitergegeben werden, heften an diesen Daten aus normativer Sicht die informationellen Kontrollrechte der betroffenen Personen (sog. Betroffenenrechte). Die Prozesse der Infrastruktur müssen gewährleisten, dass die an den Daten heftenden Betroffenenrechte auch de facto technisch und organisatorisch jederzeit auf Wunsch so gut wie möglich umsetzbar sind: die Rechte auf Auskunft und auf Herausgabe einer Kopie der Daten, auf Berichtigung, aber auch auf Widerruf der Einwilligung und Löschung der Daten.

Aus datenschutzrechtlicher Perspektive ist auch insoweit zu beachten, dass sich alle Akteure (Dateninfrastruktur, Datenerzeuger:innen, Datennutzer:innen) im Falle einer gemeinsamen Verantwortlichkeit über die Umsetzung der Betroffenenrechte verständigen müssen. Es gilt wiederum die Vorgabe des Art. 26 Abs. 1 Satz 2 DSGVO, wonach die Verantwortlichen in einer Vereinbarung transparent festlegen müssen, wer von ihnen welchen Verpflichtungen nachkommt – und zwar „insbesondere was die Wahrnehmung der Rechte der betroffenen Person angeht“.

### Umgang mit Zufalls- und Zusatzbefunden („incidental findings“ bzw. „additional findings“)

Die Frage der Rückmeldung von Zusatzbefunden wurde insbesondere aus ethischer Sicht in den letzten 10 Jahren intensiv diskutiert [13] und stellt sich nun speziell mit Blick auf die Verantwortlichkeit und Rolle der Infrastrukturen. Sie ist nicht Teil der rechtlich kodifizierten Betroffenenrechte, aber mit diesen verwandt mit Blick auf Herausforderungen der technisch-organisatorischen Umsetzung und Gewährleistung.

Bei der Nutzung von Daten zu neuen Forschungsfragen oder mit neuen Forschungsmethoden können theoretisch Informationen entdeckt werden, die für die datengebenden Patient:innen neu und von individueller gesundheitlicher Bedeutung sind. Auch für Infrastrukturen muss insoweit geklärt werden, welche Rolle diese bei der Rückmeldung von möglichen Zusatz‑/Zufallsbefunden spielen sollen. Diese Rolle muss in einer Policy entsprechend festgehalten und gegebenenfalls technisch-organisatorisch hinterlegt/vorgehalten werden.

Falls externe datennutzende Personen bei der Forschungsnutzung der Daten auf Funde treffen, die aus ihrer Sicht für das Datensubjekt, d. h. die datengebenden Patient:innen, potenziell von bedeutsamer individueller Gesundheitsrelevanz sein können, stellt sich u. a. die Frage, ob sie versuchen sollten, die Information an die Datenerzeuger:innen, die sie selbst eventuell gar nicht kennen, zu melden. Es ist auch klärungsbedürftig, welche Verantwortung die Infrastruktur mit Blick auf die mögliche Rückmeldung von Zusatzbefunden hat und welche technische und organisatorische Rolle sie dabei spielen kann oder sollte. Seitens der Infrastruktur könnte vorab gegenüber allen Datenerzeuger:innen und -nutzer:innen klar gemacht werden, dass sie einen möglichen Rückmeldeprozess von Zusatzinformationen in keiner Weise unterstützt oder technisch vorhält. Ob dies ein ethisch vertretbares Vorgehen wäre, soll hier offenbleiben.

Die Infrastruktur könnte aber auch dazu genutzt werden, Rückmeldeprozesse organisatorisch und technisch zu unterstützen. Zu beachten ist jedoch, dass sich die Rückmelde-Policies der datenerzeugenden Projekte und Konsortien stark unterscheiden können und die Patient:innen dementsprechend in ihrer Einwilligung eventuell auch zu unterschiedlichen Weisen und Verfahren der Rückmeldung von potenziellen Zusatzinformationen eingewilligt haben. Außerdem sollte eine Rückmeldung aus ethischen Gründen nicht direkt von den externen Datennutzer:innen aus der Forschung an die Patient:innen gehen, sondern immer nur nach klinischer Evaluation über die behandelnden oder andere Ärzt:innen [13].

## Orientierung am Gemeinwohl

Die NFDI-Konsortien werden von öffentlichen Geldern finanziert und haben den Auftrag, Infrastruktur für die Forschung aufzubauen. Bei öffentlich finanzierter Forschung allgemein, ganz besonders jedoch bei Gesundheitsforschung, hat die Allgemeinheit die berechtigte Erwartung, dass alle Geförderten ihr Bestes tun, um einen möglichst großen Mehrwert für die Patient:innen, das öffentliche Gesundheitssystem und die Allgemeinheit zu generieren. Konsequenzen hat dies vor allem für die Frage, mit welchem Selbstverständnis sich Forscher:innen in Infrastrukturprojekte wie NFDI einbringen und in welcher Form auch privatwirtschaftliche Unternehmen in solcherlei Projekte eingebunden werden sollen.

### Dienst an der Forschungsgemeinschaft

In den NFDI-Konsortien haben sich Forscher:innen aus dem Bereich der öffentlich-akademischen Wissenschaft zusammengetan, um finanziert durch Drittmittel IT-Infrastruktur aufzubauen, die einer Forschungscommunity für datengetriebene Forschung zur Verfügung stehen soll: Forscher:innen bauen eine Dateninfrastruktur auf, Vertreter:innen von IT- und Computerwissenschaften entwickeln Interfaces und Softwaretools, Rechtswissenschaftler:innen entwickeln Datenschutzkonzepte gemeinsam mit den Forscher:innen und Betreiber:innen, Ethiker:innen schreiben an Informations- und Aufklärungsdokumenten mit, andere entwickeln Community-weite Standards für Forschungsdatensätze und Metadaten usw.

Es stellt sich die Frage, aus welchen Antrieben Forscher:innen diese Aufgaben übernehmen und welche Interessen sie dabei verfolgen sollten. Kernaufgabe von Forscher:innen war bisher klassischerweise die Entwicklung und Publikation neuer wissenschaftlicher Methoden und Erkenntnisse. Der engagierte Einsatz in einem Konsortium mit all seinen Arbeitsgruppen und Abstimmungsrunden zum Aufbau einer Forschungsdateninfrastruktur für eine bestimmte Wissenschaftscommunity ist nur sehr begrenzt dazu geeignet, neue publizierbare wissenschaftliche Erkenntnisse zu gewinnen. Die Forscher:innen selbst müssen sich der herrschenden widerstreitenden Erwartungen an sie – Aufbau von Infrastruktur versus Publizieren – bewusst sein. Ihre Institutionen und andere Einrichtungen des Wissenschaftssystems sollten sich über zusätzliche und passende Formen der Leistungsanerkennung Gedanken machen.

Unabhängig davon haben Forscher:innen, die sich in den Konsortien engagieren und hierfür Drittmittel empfangen, entsprechend der Aufgabenstellung und den Zielen der NFDI die Pflicht und Verantwortung gegenüber der geldgebenden Öffentlichkeit und der jeweiligen Wissenschaftscommunity, sich beim Aufbau der Forschungsinfrastruktur am Interesse der gesamten Wissenschaftscommunity zu orientieren und etwaige Partikularinteressen den Anliegen und Interessen der gesamten Forschungscommunity unterzuordnen. Alle involvierten Forscher:innen müssen etwa im Bereich datenintensiver Biomedizin Infrastruktur aufbauen, die sich technisch, wissenschaftlich und organisatorisch auf sinnvolle und harmonische Weise mit anderen großen Infrastrukturprojekten und Initiativen ergänzt, anstatt aus Partikularinteressen Silolösungen oder Doppelstrukturen zu schaffen.

### Einbeziehung von privatwirtschaftlichen Unternehmen

Klärungsbedürftig ist auch, inwieweit sich die Maxime einer gemeinwohlorientierten Forschung mit der Einbindung auch privatwirtschaftlicher Unternehmen vereinbaren lässt. Private forschende Unternehmen spielen generell eine große Rolle für die Forschung in Deutschland, speziell mit Blick auf die Entwicklung neuer Therapien. Dennoch sehen Patient:innen und Bürger:innen es kritischer als bei öffentlich-akademischen Forschungseinrichtungen, dass privatwirtschaftliche Unternehmen Zugang zu und Nutzungsrechte an ihren Daten haben sollen [14]. Aus ethischer Perspektive ist eine Einbeziehung von privatwirtschaftlichen Unternehmen zu medizinischen Forschungszwecken wünschenswert, *wenn* sie die Rechte und Sorgen der Patient:innen (und Bürger:innen) angemessen berücksichtigt *und* auch einen klaren Mehrwert für das öffentliche Gesundheitswesen und letztlich das Gemeinwohl schafft.

Soweit eine Forschungsdatenverarbeitung einwilligungsbasiert erfolgt, hängt es zuallererst an den konkreten Inhalten der verwendeten Einwilligung und dem Willen der Datenerzeuger:innen, ob personenbezogene Daten auch an die Privatwirtschaft zu Forschungszwecken weitergegeben werden. Auch wenn die Einwilligung unmittelbar nur den Datenerzeuger:innen gegenüber erteilt wird, kann diese dann zugleich auch im Rahmen der Infrastruktur eine rechtswirksame Legitimationsgrundlage für die Weitergabe der Daten darstellen. Um insoweit sicherzustellen, dass es sich auch tatsächlich um eine *freiwillige und informierte* Einwilligung handelt, können seitens der Infrastruktur etwa modellartige Textbausteine für die Patientenaufklärung und Einwilligung zur Verfügung gestellt werden. Alternativ kann eine Liste ethischer Kriterien oder „points to consider“ aufgestellt werden, die erfüllt sein sollen, wenn Privatunternehmen Zugang zu und Nutzungsrechte an Daten erhalten wollen.

Außerhalb von einwilligungsbasierten Lösungen stellt sich die Frage, ob und inwieweit auch privatwirtschaftliche Unternehmen als forschende Institutionen unter das rechtliche Forschungsprivileg fallen und damit unter deutlich erleichterten Bedingungen personenbezogene Daten verarbeiten können. Die DSGVO versteht ausweislich ihres Erwägungsgrunds 159 den Begriff der wissenschaftlichen Forschung weit, dieser soll u. a. auch die privat finanzierte Forschung umfassen. Dem entspricht das weite Verständnis von Wissenschaftsfreiheit nach der Rechtsprechung des Bundesverfassungsgerichts, wonach sich diese auf „jede wissenschaftliche Tätigkeit“ erstreckt, „das heißt auf alles, was nach Inhalt und Form als ernsthafter planmäßiger Versuch zur Ermittlung von Wahrheit anzusehen ist“ [15, 16].

Andererseits kann aber nicht jede Form einer Analyse und Aufbereitung von Daten auch schon eine datenschutzrechtliche Sonderrolle für sich beanspruchen, insbesondere dann, wenn es in erster Linie um eine kommerziell motivierte Datenverarbeitung geht. Die Privilegierung einer Datenverarbeitung zu Forschungszwecken lässt sich vielmehr nur dann rechtfertigen, wenn die damit einhergehenden Einschränkungen des Grundrechts auf informationelle Selbstbestimmung durch andere Belange des Allgemeinwohls kompensiert werden.

Entscheidend muss daher für eine datenschutzrechtliche Privilegierung auch privatwirtschaftlicher Forschung sein, dass diese die Kernmerkmale unabhängiger wissenschaftlicher Forschung erfüllt: Ist eine Transparenz des Forschungsprozesses und der Ergebnisse gewährleistet? Wird das Ziel eines Erkenntnisgewinns auch im Allgemeininteresse nicht von sachfremden Erwägungen überlagert [11, 17 Rn. 13, 18, S. 19 ff.]?

Im Einzelnen sind hier noch viel konzeptionelle Differenzierungsarbeit und normative Reflexion zu leisten.^6^ Aus ethischer Sicht kommt eine ganze Reihe von Bedingungen und Maßnahmen in Betracht, die zu beachten sind, wenn privatwirtschaftliche Unternehmen für die sekundäre Forschungsnutzung Zugang zu Patientendaten aus dem öffentlichen Gesundheitswesen erhalten sollen: spezielle Aufklärung, spezielle Einwilligungs- und Widerspruchsmöglichkeiten, enge Begrenzung auf Forschung mit Potenzial und Ziel der Verbesserung der klinischen Versorgung, Aufwandsentschädigung an die öffentlich finanzierte Infrastruktur, ein unbürokratisch zu aktivierender Haftungsfond usw. [20, 21].

## Partizipation, Transparenz und Wissenschaftskommunikation

Für die Infrastrukturen gelten die ethischen Anforderungen der Partizipation, Transparenz und guten Kommunikation. Diese vornehmlich ethischen Anforderungen sind u. a. auch unabdingbar, um das Vertrauen der Öffentlichkeit und aller anderen Stakeholder in die Infrastrukturen und in die angestrebten Verwendungsweisen der Daten zu Forschungszwecken zu etablieren und aufrechtzuerhalten.^7^

Schon bei der Entwicklung von Werkzeugen, Prozessen und normativer Governance durch Infrastrukturen sollten die jeweils besonders betroffenen Stakeholder partizipieren können. Ebenso sollten sich im Rahmen von Infrastrukturen für personenbezogene Daten (Vertreter:innen von) Patient:innen und Bürger:innen an den etablierten Strukturen und Entscheidungsgremien wirksam, dauerhaft und nachhaltig beteiligen können.

Über alle wesentlichen Aspekte der Infrastruktur (Ziele, Gremien, involvierte Personen und Institutionen, Verfahren, Gelder, Interessenkonflikte, Aufsichtsstrukturen, Leistungen, Ergebnisse, Misserfolge, ermöglichter Wissensgewinn usw.) sollte nach außen hin transparent informiert werden. Auch darüber, wie Patient:innen, Bürger:innen oder andere Stakeholder, z. B. externe Wissenschaftler:innen, in die anfängliche Entwicklung bestimmter Prozesse und Verfahrensstandards und, wenn diese einmal entwickelt sind, in deren Durchführung und Betrieb involviert waren und sind.

Dabei sollten proaktiv auch Patient:innen, Bürger:innen und eine breitere Öffentlichkeit in laienverständlicher und niederschwelliger Weise über verschiedene Kanäle und Medien angesprochen und informiert werden: insbesondere über den Nutzen der Infrastrukturen und unterstützten Forschung für die Gesellschaft, über die Aspekte, die für Patient:innen oder Bürger:innen eventuell besonders sensibel oder besorgniserregend sind, sowie über Möglichkeiten, sich selbst in den Aufbau und Betrieb der Infrastrukturen einzubringen, im Sinne der Partizipation.

## Fazit und Ausblick

Das Vertrauen der Stakeholder ist eine zentrale Voraussetzung für den erfolgreichen Aufbau und Betrieb der Infrastrukturen. Durch die Synchronisierung der derzeit laufenden Fördermaßnahmen rund um die Medizininformatik-Initiative (MII), das Netzwerk Universitätsmedizin (NUM) und nicht zuletzt die Nationale Forschungsdateninfrastruktur (NFDI) werden im großen Stil Prozesse und Infrastrukturen entwickelt, getestet und ausgerollt, die erstmals die Sichtung, Beantragung und Nutzung großer Datenmengen ermöglichen.

Diese Nutzung birgt die Herausforderung, das Vertrauen und die Akzeptanz der potenziellen Datengeber:innen gewinnen und erhalten zu müssen, d. h. letztlich der Patient:innen und Bürger:innen. Diese bringen der akademischen Forschung einerseits einen großen Vertrauensvorschuss entgegen. Andererseits sind mit Aufbau und Betrieb der geplanten Infrastrukturen auch Aspekte betroffen, denen Teile der Bevölkerung mit Bedenken gegenüberstehen und die auch aus ethischer und rechtlicher Perspektive komplex sind, wie z. B. Fragen der Aufklärung und Einwilligung, die Nutzung personenbezogener Daten wie Gesundheits-, genetischer bzw. genomischer Daten, die Einbeziehung von privatwirtschaftlichen Unternehmen oder die Kooperation mit Forscher:innen im Ausland mit niedrigeren Datenschutzstandards. Diese Herausforderungen sind im Dialog mit den gegenwärtigen und potenziellen Datengeber:innen und den Datennutzer:innen und methodisch in Austausch und Zusammenarbeit zwischen der biomedizinischen Informatik, der Ethik und den Rechtswissenschaften zu lösen.

## Footnotes

1 Siehe zum Hintergrund die Informationen auf der offiziellen Website der NFDI: https://www.nfdi.de/verein/#historie.

2 Wenn hier und im Folgenden von „der Dateninfrastruktur“ und ihren Verantwortlichkeiten die Rede ist, ist damit (datenschutz-)rechtlich im Sinne des Art. 4 Nr. 7 Datenschutz-Grundverordnung (DSGVO) „die natürliche oder juristische Person, Behörde, Einrichtung oder andere Stelle“, die hinter dieser Infrastruktur bzw. dem Konsortium steht, gemeint und ethisch eine institutionelle Einrichtung der öffentlich-akademischen Wissenschaft.

3 Zuletzt kritisch zum *Broad Consent* als Legitimationsgrundlage für eine Forschungsdatenverarbeitung aus rechtlicher Warte [5, 6].

4 Zu einem Überblick über verschiedene Einwilligungsmodelle s. [7]. Zu den technischen, datenschutzrechtlichen und ethischen Herausforderungen (bei der Umsetzung) der sogenannten dynamischen Einwilligung (Dynamic Consent) s. [8].

5 Zum weiten Verständnis der Forschungsfreiheit s. unten „Einbeziehung von privatwirtschaftlichen Unternehmen“.

6 S. aktuell zur Frage der Privilegierung privatwirtschaftlicher Forschung im Rahmen des European Health Data Space [19].

7 Siehe dazu u. a. das forschungspolitische Grundsatzpapier des Bundesministeriums für Bildung und Forschung (BMBF) zur Partizipation https://www.bmbf.de/bmbf/shareddocs/downloads/files/bmbf_grundsatzpapier_partizipation_barrierefrei.pdf?__blob=publicationFile&v=2.
